# Novel Form of Curcumin Improves Endothelial Function in Young, Healthy Individuals: A Double-Blind Placebo Controlled Study

**DOI:** 10.1155/2016/1089653

**Published:** 2016-08-17

**Authors:** Jonathan M. Oliver, Lee Stoner, David S. Rowlands, Aaron R. Caldwell, Elizabeth Sanders, Andreas Kreutzer, Joel B. Mitchell, Martin Purpura, Ralf Jäger

**Affiliations:** ^1^Department of Kinesiology, Texas Christian University, TCU Box 297730, Fort Worth, TX 76129, USA; ^2^School of Sport and Exercise, Massey University, 63 Wallace Street, Wellington 6021, New Zealand; ^3^Increnovo LLC, 2138 E. Lafayette Place, Milwaukee, WI 53202, USA

## Abstract

Curcumin, a turmeric extract, may protect against cardiovascular diseases by enhancing endothelial function. In this randomized controlled double-blind parallel prospective study, fifty-nine healthy adults were assigned to placebo, 50 mg (50 mg), or 200 mg (200 mg) curcumin, for 8 weeks. The higher curcumin (200 mg) supplementation produced a dose-mediated improvement in endothelial function measured by flow-mediated dilation (FMD). The outcome was a clinically substantial 3.0% increase (90% CI 0.7 to 5.3%, *p* = 0.032; benefit : harm odds ratio 546 : 1) with the 200 mg dose, relative to placebo. The 50 mg dose also increased FMD relative to placebo by 1.7% (−0.6 to 4.0%, *p* = 0.23; 25 : 1), but the outcome was not clinically decisive. In apparently healthy adults, 8 weeks of 200 mg oral curcumin supplementation resulted in a clinically meaningful improvement in endothelial function as measured by FMD. Oral curcumin supplementation may present a simple lifestyle strategy for decreasing the risk of cardiovascular diseases. This trial was registered at ISRCTN registry (ISRCTN90184217).

## 1. Introduction

Cardiovascular diseases (CVD) are often asymptomatic and can begin as early as childhood [1]. A putative mechanism leading to CVD is damage to the vascular endothelium, a monolayer of cells which releases antiatherosclerotic molecules, most notably including nitric oxide [2]. Endothelial function can be measured as a decreased flow-mediated dilation (FMD) response, using occlusion of the brachial artery and the subsequent dilation response as an indicator of vascular function [1, 3]. Lifestyle behaviours which enhance antioxidative status and preserve nitric oxide bioavailability may protect against endothelial dysfunction [4–6].

Curcumin, the major yellow pigment extracted from turmeric, a commonly used spice in India and Southeast Asia, has been demonstrated to be cardioprotective [7] and promote antioxidative and anti-inflammatory pathways [8, 9]. Commercially available natural curcumin is a mixture of three curcuminoids: curcumin (71.5%), demethoxycurcumin (19.4%), and bisdemethoxycurcumin (9.1%) [10]. Thus, curcumin may present a simple, cost-effective, and nonpharmaceutical preventive and therapeutic approach for preserving endothelial function. However, only two known studies have investigated the effects of curcumin, or curcuminoids, on endothelial function, one reporting the reversal of homocysteine-induced endothelial dysfunction in porcine coronary arteries [11] and the other reporting that 8-week supplementation or aerobic exercise similarly enhanced endothelial function in postmenopausal older women (mean 60 y) with low baseline FMD (<3%) [12].

No known studies have assessed whether chronic curcumin supplementation can improve endothelial function in an apparently healthy population. Therefore, the purpose of this study was to determine whether 8 weeks of low- or high-dose oral curcumin supplementation enhances FMD, the standard measure of endothelial function, in apparently healthy individuals. We hypothesized that curcumin supplementation would result in a worthwhile clinical effect (absolute 1% improvement in FMD) [13] in a dose-dependent manner.

## 2. Material and Methods

### 2.1. Subject Population

This study was conducted according to the Declaration of Helsinki guidelines and registered with ISRCTN registry (ISRCTN90184217). All procedures involving human subjects were approved by the Institutional Review Board of Texas Christian University for use of human subjects in research (1410-105-1410). Written consent was obtained from all subjects.

The health status and activity levels of potential participants were determined by completion of a medical history form and an activity record. Eligibility criteria included nonsmoking men and women aged 19 to 29 years with no musculoskeletal, medical, or metabolic contraindications to exercise and low to moderately trained, defined as meeting the current American College of Sports Medicine guidelines of at least 150 min of moderate aerobic activity or 75 min per week of vigorous aerobic activity per week for the past three months [14]. Exclusion criteria included women who were pregnant or lactating, participation in another clinical trial or consumption of investigational product within the previous thirty days, receiving regular treatment with anti-inflammatory/analgesic/antioxidant drugs in the previous month, or use of any ergogenic aid during the nine-week period prior to recruitment.

### 2.2. Study Design

A randomized, placebo controlled, double-blind parallel design was employed to examine the effect of high and low doses of a novel form of curcumin on endothelial function in reportedly healthy young adults with no known risk for cardiovascular disease. Prior to experimental testing, subjects completed medical history, exercise, and demographic questionnaires. A pregnancy test was administered for females. On the day of experimental testing, subjects arrived to the laboratory fasted (>10 h), having refrained from all physical activity outside of daily living for the previous 72 h for venous blood sampling. Height and body weight were determined to the nearest 0.1 cm and 0.1 kg, respectively, using a stadiometer (Seca, Chino, CA) and self-calibrating digital scale (Seca, Chino, CA) with subjects in socks or bare feet. Subsequently, endothelial function (flow-mediated dilation, FMD) was assessed followed by maximal aerobic capacity (${\dot{V}O}_{2max}$). Participants were then matched according to body mass and randomly assigned to ingest, in a double blind manner, either 50 mg curcumin (50 mg), 200 mg curcumin (200 mg), or placebo. Participants were advised to maintain their current diet and exercise program for the duration of the 8-week supplementation period. At the conclusion of the 8-week supplementation (56 days), all experimental testing procedures were repeated.

### 2.3. Supplementation

The investigational product (CurcuWIN®, OmniActive Health Technologies Ltd., Mumbai, India) contained turmeric extract (20–28%), a hydrophilic carrier (63–75%), cellulosic derivatives (10–40%), and natural antioxidants (1–3%) [15]. During the consent and familiarization process, subjects were educated on which foods contained turmeric and asked to avoid those foods for the duration of the study while maintaining their normal diet. Participants were assigned to placebo (corn starch), low-dose (50 mg curcumin = 250 mg CurcuWIN; 50 mg), or high-dose (200 mg curcumin = 1,000 mg CurcuWIN; 200 mg) curcumin supplementation in the form of CurcuWIN containing the three curcuminoids: curcumin (71.5%), demethoxycurcumin (19.4%), and bisdemethoxycurcumin (9.1%) [10]. The day following baseline testing, participants were asked to ingest one equal dose capsule with breakfast, lunch, and dinner, 3 capsules per day in total. To aid compliance, participants were provided with the capsules on a weekly basis in a daily pill container and were asked to return the empty containers. An adverse side effects questionnaire was also completed when receiving capsules. Compliance was set at ≥80%.

### 2.4. Fasting Venous Sampling

Prior to the FMD procedure, a venous blood sample was obtained after an overnight fast (>10 h). Upon arrival to the laboratory, participants lay in the supine position while the antecubital area was sterilized using standard sterile phlebotomy procedures and a venous blood sample was taken from an antecubital vein. Samples were sent to a commercial laboratory (LabCorp, Fort Worth, TX) to perform safety analysis, which included a complete blood count and metabolic panel (albumin, alkaline phosphatase, alanine aminotransferase, aspartate aminotransferase, bilirubin, blood urea nitrogen, calcium, chloride, creatinine, gamma-glutamyl transpeptidase, globulin, glucose, iron, lactate dehydrogenase, phosphorous, potassium, protein, sodium, and uric acid).

### 2.5. Flow-Mediated Dilation (FMD)

Endothelial function was assessed using the standard flow-mediated dilation (FMD) test [16], which has been demonstrated to be at least partially nitric oxide dependent [17, 18]. Participants were examined by the same sonographer in a sound-isolated, temperature-controlled room following an overnight fast (>10 h). The Typical Error Estimate (TEE) [19, 20] on a random sampling of subjects from the placebo group at two time points was 0.73. Females on oral contraceptives reported to the lab on days 10–12 of their menstrual cycle for baseline testing. Females not on oral contraceptives reported to the lab on days 4–6 of their menstrual cycle for baseline testing. Postsupplementation testing was performed on day 56 for all female subjects coincident with the baseline testing cycle stage [16]. Brightness-mode ultrasound measurements were made using an Acuson Aspen Ultrasound system (Mountain View, CA) equipped with a 7–10 MHz linear-array transducer (L10) at a sampling rate of 20–25 Hz. A custom-built arm rest was used to prevent movement during the procedure and the probe was locked into place using a custom-built probe-holding device. A standard adult blood pressure cuff was wrapped around the right forearm approximately 10 cm distal to the antecubital space. Following a quiet rest period in the supine anatomical position of at least 10 min, the right brachial artery diameter was scanned (the longitudinal plane 3–8 cm proximal to the antecubital fossa along the medial aspect of the arm). Care was taken to ensure that the vessel clearly extended across the entire imaging plane to minimize the likelihood of skewing the vessel walls. Following collection of baseline images (3 × 8 second clips), the blood pressure cuff was inflated to 50 mmHg above resting systolic blood pressure for 5 minutes. Imaging of the vessel diameter resumed 30 seconds prior to cuff deflation and continued for 3 minutes after deflation (7 × 30 second clips). Baseline and postdeflation vessel diameters were analyzed using semiautomated brachial analyzer software (Medical Imaging Applications, LLC, Coralville, IA). FMD was calculated as (maximum diameter − baseline diameter)/baseline diameter ×100, where the maximum diameter represents the maximum diameter after 5-minute distal ischemia.

### 2.6. Maximal Aerobic Capacity (${\dot{V}O}_{\text{2max}}$)

Maximal aerobic capacity was determined using an incremental treadmill test to exhaustion. Throughout the test, respiratory gas exchange was measured using an open-circuit gas analysis system (True One, Parvo Medics, Sandy, Utah), and heart rate was monitored using a telemetry system (Polar Electro E600, Polar Electro Inc., Lake Success, New York). This test was performed to ensure activity regularly performed did not result in a likely clinical improvement in endothelial function.

### 2.7. Data Presentation and Transformation

The effect of curcumin dose on outcomes was estimated with a mixed model analysis of variance (Proc Mixed, SAS 9.4, Cary, NC). The primary outcome was the treatment*∗*time interaction, which is a within-model analysis of postbaseline change scores to adjust for baseline variability. The addition of sex (male, female) and ethnicity (Asian, White, Latino, or African-American) as binary covariates resulted in the best model fit parameters. The subject term was the random effect, with sex = female also placed as an additional random effect following variance analysis. Estimates for effect of treatment were presented as means with uncertainty as 90% confidence interval (CI) [21].

### 2.8. Statistical Inference

A modified posterior Bayesian approach, termed magnitude-based inference, was employed to infer outcome effects to a large sample of the population [22, 23]. The approach emphasizes clinical effect size and likelihood, avoiding some pitfalls of the hypothesis-testing based *p* value based approach to inference [21, 24]. Accordingly, the threshold for smallest worthwhile clinical change in FMD was 1% [25]; for other parameters, we used Cohen's *d* standardized difference (0.2 × baseline SD) [23], unless otherwise noted. The probability that a contrast was at least greater than the clinical threshold was 25–75% possible, 75–95% likely, 95–99.5% very likely, and >99.5% almost certain [23]. In the case where the majority (>50%) of the CI lie between the thresholds for positive and negative substantiveness, the effect was qualified trivial (negligible) with the respective probabilities as above [26]. The terms* benefit, trivial (negligible),* and* harm* refer to the most likely directional outcome, relative to the smallest effect threshold. The terms* unclear *and* inconclusive* refer to outcomes where the likelihood of both benefit and harm exceeded 5%. The likelihood of a clinical benefit of intervention was expressed as the benefit : harm odds ratio, with 66 : 1 as the smallest adoption threshold [23].

## 3. Results

### 3.1. Demographics and Safety Measures

Baseline demographics partitioned by experimental treatment group are shown in Table 1. Differences in parameters between groups (mixed model analysis of variance) were unclear or trivial (*p* > 0.12), except for a −6.5 mmHg higher (90% CL: −2.5, −10.5 mmHg, *p* = 0.009) diastolic blood pressure in the 200 mg versus 50 mg curcumin groups. Safety data obtained from venous blood sampling (complete blood count and metabolic panel) were within normal range and no adverse events were observed during 8-week supplementation.

### 3.2. Maximal Aerobic Capacity (${\dot{V}O}_{\text{2max}}$)

A small most likely trivial improvement in ${\dot{V}O}_{2max}$ was observed in all treatment groups (mean improvement; ±90 CI; PLA, 1.3; ±1.7 mL·kg·min^−1^; 50 mg, 0.11; ±1.9 mL·kg·min^−1^; 200 mg, 1.7; ±1.8 mL·kg·min^−1^) indicating that the exercise performed over the 8-week intervention was not sufficient to induce significant adaptations (smallest worthwhile clinical change 5 mL·kg·min^−1^), specifically endothelial function.

### 3.3. Flow-Mediated Dilation

**Figure 1:**
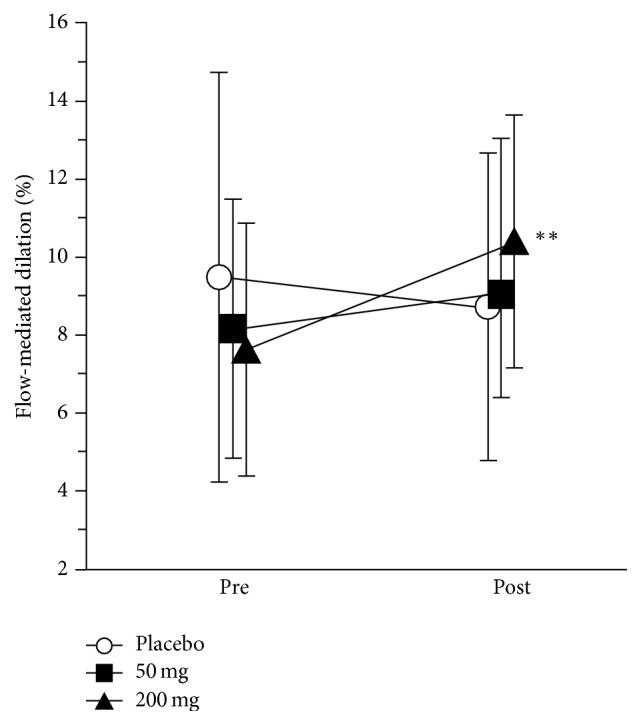
Effect of 56 day curcumin supplementation on flow-mediated dilation (%). Data are raw means and SD for the pre- (baseline) and posttesting time points. The magnitude threshold for the smallest change was 1%. The *∗∗* symbols indicate the likely substantial increase in the 200 mg dose, relative to placebo (Table 2).

The effect of 56 days of curcumin supplementation on FMD is shown in Figure 1. The supplement resulted in clinically substantial dose-mediated increases in mean FMD of between 1.7 and 3.0 percent (Table 2), with the 200 mg dose contrast relative to placebo being statistically significant (*p* < 0.032) and clinically meaningful (>1%) with high likelihood of clinical benefit and odds ratio (Table 2). While the increase in FMD with the 50 mg dose relative to placebo was also higher than the smallest clinical threshold, the outcome was less clear (Table 2).

## 4. Discussion

The main finding of this study was a clinically substantial 3.0% increase in FMD following 8 weeks of high-dose (200 mg) curcumin supplementation and a possible 1.7% improvement following low-dose (50 mg) supplementation. Considering FMD is the standard test of endothelial function, these findings provide preliminary evidence to support the notion that chronic curcumin supplementation may decrease the risk of CVD in persons who are apparently healthy.

The current findings support a previous study by Akazawa et al. [12] which reported improved FMD in postmenopausal women following an 8-week intervention of 150 mg/d theracurcumin. In contrast to the current study, the participants in the aforementioned study were postmenopausal and older (mean 60 y) and had a low baseline FMD (<3%)—indicating heightened risk for CVD. The current study, to the best of our knowledge, is the first to report that curcumin may enhance endothelial function in young, apparently healthy participants without established CVD risk factors.

While further study is required to elucidate the mechanism by which curcumin improves endothelial function, antioxidative and anti-inflammatory actions are plausible [8, 9]. Curcumin suppresses inflammation by inhibiting I*κ*B Kinase (IKK) signaling complex thereby preventing the activation of nuclear factor-kappa B (NF-*κ*B) [27–30]. Curcumin also reduces inflammatory signaling by inhibiting the binding of activator protein-1 (AP-1) to DNA [27] and the production of cyclooxygenase-2 enzyme (COX-2) [31]. Additionally, curcumin may protect against oxidative stress through the production of endothelial hemeoxygenase, an inducible stress protein that degrades heme to the vasoactive molecule and the antioxidant biliverdin [32].

Subsequent curcumin supplementation studies should ensure that the supplement is of a sufficient dose and a suitable quality. Poor solubility, low absorption from the gut, rapid metabolism, and systemic elimination are all major limitations to the therapeutic potential of curcumin [33]. The curcumin used in the current study has been shown to have a 45.9-fold higher absorption over standard curcumin [15]. This may explain why, even though previous studies have reported little to no appearance of curcumin in the circulation following high-dose (500–12,000 mg) supplementation [34], the novel form used in the current study improved FMD even though the highest dose was limited to 200 mg curcumin.

## 5. Conclusions

Findings from this study suggest that chronic supplementation with 200 mg per day curcumin enhances endothelial function as measured by FMD in apparently healthy young subjects. Oral curcumin supplementation may present a simple lifestyle strategy for decreasing the risk of cardiovascular diseases in individuals who are apparently healthy.

## Figures and Tables

**Table 1:** Baseline demographics of the experimental cohort partitioned by treatment group.

Parameter	Treatment group	Mean + SD
Age (y)	Placebo	20.8 ± 1.8
	50 mg	21.2 ± 2.3
	200 mg	21.7 ± 2.4

Stature (cm)	Placebo	170.6 ± 8.2
	50 mg	173.7 ± 9.3
	200 mg	169.2 ± 9.9

Weight (kg)	Placebo	69.8 ± 11.3
	50 mg	73.2 ± 14.9
	200 mg	66.9 ± 14.6

Body mass index (kg·m^−2^)	Placebo	23.9 ± 2.6
	50 mg	24.1 ± 3.4
	200 mg	23.1 ± 3.1

Systolic blood pressure (mmHg)	Placebo	116.9 ± 7.3
	50 mg	115.7 ± 7.5
	200 mg	118.3 ± 6.0

Diastolic blood pressure (mmHg)	Placebo	74.6 ± 6.6
	50 mg	71.4 ± 8.3
	200 mg	77.8 ± 6.6

Resting HR (beats·min^−1^)	Placebo	73.7 ± 8.4
	50 mg	74.8 ± 7.5
	200 mg	74.9 ± 10.4

Maximal oxygen consumption (mL·kg·min^−1^)	Placebo	41.6 ± 6.0
	50 mg	43.7 ± 7.1
	200 mg	44.1 ± 7.6

Sample size per group: PLA, 21; 50 mg, 19; 200 mg, 19.

**Table 2:** A statistical summary of the effect of 56-day curcuminoid supplementation on flow mediated dilation (%).

Contrast	Estimate	Confidence interval (90%)		*p* value	Clinical inference statistics		
		Upper	Lower		Likelihood (%) benefit/trivial/harm^c^	Qualitative^c^	Benefit odds^c^
Time effect^a^							
Placebo	−0.3	1.3	−1.9	0.769			
50 mg	1.4	3.1	−0.3	0.174			
200 mg	2.7	4.3	1.1	0.007			

Treatment *∗* time effect^b^							
50 mg-Placebo	1.7	4.0	−0.6	0.234	68.6/28.6/2.8	↑ possible	25 : 1
200 mg-Placebo	3.0	5.3	0.7	0.032	92.8/7.0/0.2	↑ likely	546 : 1
200 mg-50 mg	1.3	3.7	−1.0	0.349	59.2/35.8/4.9	↑ possible	12 : 1

^a^Post-pre intervention.

^b^For example, 50 mg post-pre–200 mg post-pre.

^c^The smallest worthwhile clinical change is 1%. The probability that a contrast was at least greater than the clinical threshold was 25–75% possible, 75–95% likely, 95–99.5% very likely, and >99.5% almost certain. The terms *benefit*, *trivial*, and *harm* also denoted by symbols ↑, *↔*, and ↓, respectively, refer to the most likely directional outcome relative to the smallest effect threshold. *Unclear* refers to outcomes where the likelihood of both benefit and harm exceeded 5%. The clinical adoption threshold was expressed as a benefit: harm odds ratio >66 : 1.
